# Supplementary material for: SPINK2 Protein Expression Is an Independent Adverse Prognostic Marker in AML and Is Potentially Implicated in the Regulation of Ferroptosis and Immune Response
Source: Int J Mol Sci. 2023 Jun 2;24(11):9696. doi: 10.3390/ijms24119696 (PMC10253579; doi:10.3390/ijms24119696)
Supplement: Supplementary file 1 [file ijms-24-09696-s001.zip › Supplementary_edited_02-06-2023.pdf]

SPINK2 protein expression is an independent adverse prognostic marker in AML, and is potentially implicated in the regulation of ferroptosis and immune response

Supplementary information & figures

Supplementary methods and information

Identification of *SPINK2* overexpression in AML and functionally defined LSC fractions

The Oncomine [1] database was used to initially compare microarray gene expression data between AML samples (N=831) and normal bone marrow (NBM) samples (N=141) in 4 independent datasets (GSE7186, GSE13164, GSE13159, GSE995) generating a list of differentially expressed genes. The top-50 genes by median-ranked analysis were further selected. From these 50 genes, only those were further selected which were (i) not well characterized in AML, and (ii) part of a recently generated LSC gene signature from Ng *et al* [2]. Four genes were selected by these criteria: *SHANK3*, *GPSM1*, *FSCN1* and *SPINK2*. Median expression of the 4 genes was then compared between sorted CD34$^+$ AML cells (n=46) and sorted CD34$^+$ NBM cells (n=31) in the GSE30029 dataset [3]. Of the 4 genes, *SPINK2* had significantly highest fold-change (*SPINK2*: 2.34, p=0.0065; *FSCN1*: 1.53, p=0.004; *GPSM1*: 1.37, p=0.086; *SHANK3*: 1.29, p=0.19). Furthermore, median expression of these genes was also compared between functionally defined LSC-enriched (LSC$^+$, n=25) and LSC-depleted (LSC$^-$, n=29) populations in the Eppert *et al* dataset (GSE30377) [4]. *SPINK2* and *FSCN1* were significantly upregulated in LSC$^+$ vs. LSC$^-$ populations (*SPINK2*: 1.653 vs. -0.2122, *P*=0.032; *FSCN1*: 0.2649 vs. -0.3189, *P*=0.034), whereas there was no data available for the other 2 genes (*SHANK3, GPSM1*). In the Ng *et al* dataset, *SPINK2* was increased approximately 4-fold in the functionally defined LSC fraction vs non-LSC fraction, while *FSCN1* was increased around 2.5-fold (Data obtained from original study, extended data table 1 "List of 104 DE LSC genes") [2]. Based upon these initial observations, *SPINK2* was chosen for further analysis. From the initial Oncomine analysis, *SPINK2* expression was significantly increased more than 2-fold in AML vs. NBM in all 4 datasets. Further Oncomine analyses of relative *SPINK2* gene expression among 3,248 leukaemia patients (AML, CML, ALL, CLL) demonstrated relatively high *SPINK2* expression specifically in AML patients.

Public datasets used for validation of clinical findings in adult and pediatric AML

TCGA-LAML (N=200)

RNA Sequencing data was available for 173 out of 200 patients included into The Cancer Genome Atlas (TCGA) adult AML study [6]. *SPINK2* RPKM expression values were downloaded for each patient from cBioPortal (https://www.cbioportal.org/) and detailed clinical and mutational information for 200 patients was kindly provided by Prof. T Ley (timley@wustl.edu) in January 2019. A value of 1 was added to each RPKM value before log2-transformation was performed. Patients were dichotomized into higher and lower *SPINK2* expression groups by the median to analyse the correlation of *SPINK2* expression with cytogenetic and mutational status. Out of the 173 patients, 58 patients were excluded from the survival analysis because they either were of FAB M3 subtype (N=16) or received induction with therapeutics not involving the standard DA 7+3 regimen backbone (N=36) or had OS <1 month(N=4) or had incomplete data (N=2). This left a more homogeneously treated subgroup of 115 patients. Of note, only OS data was available for analysis. For survival analysis, patients were dichotomized into high and low *SPINK2* groups based upon the median of *SPINK2* expression. For the pairwise multivariate Cox analysis comparing LSC gene expression signatures and *SPINK2* expression, three previously published LSC gene expression signatures (Ng [2], Gentles [7] and Eppert [4]) were used. The scores of each patient sample were calculated using the gene signatures as described in the respective publications.

OHSU BEAT-AML, N=672

RNA-Sequencing data for *SPINK2* was available for 405/672 patients included into the BEAT AML study [8]. Of these, patients not having a diagnosis of AML (N=13) were excluded, leaving 392 patients with complete mutational data for analysis. *SPINK2* RPKM expression values were downloaded for each patient from cBioPortal (https://www.cbioportal.org/) in July 2020, including mutational, cytogenetic and clinical information for each patient. For analysis of *SPINK2* and chemotherapy

response, 180 patients were analysed since they (i) were without a diagnosis of AML with myelodysplasia-related changes or therapy-related AML, (ii) were treated on standard induction regimens involving cytarabine and anthracycline backbones, and (iii) had available data on treatment response.

Verhaak (GSE6891, N=537)

This dataset [9] comprises of 537 adult *de novo* AML patients ≤60 years of age treated according to the protocols of the Dutch-Belgian Haematology-Oncology Cooperative Group (available at www.hovon.nl). Log-transformed microarray gene expression data and other relevant clinical data available for 458 patients were downloaded from NCBI GEO database and/or kindly provided by direct correspondence with Prof P.J.M Valk (p.valk@erasmusmc.nl) in September 2018. After excluding 17 patients with MDS and 24 patients with FAB M3, 417 patients were included for clinicopathological analysis. Patients were dichotomised into high and low *SPINK2* groups by the median.

Balgobind Pediatric AML (GSE17855, N=237)

Microarray gene expression data of this cohort [10] were downloaded from NCBI GEO, and clinical data of the patients were kindly provided by Prof Monique L. den Boer (M.L.denBoer@prinsesmaximacentrum.nl) in December 2019. Only 193 out of 237 were included into the survival and treatment-response analysis after exclusion of patients having no survival data (N=16), patients with OS less than 1 month (N=14), and patients with t(15;17) AML (N=14). More detailed information of this patient cohort can be found in the original article [10].

TARGET-AML (pediatric), N=235

Freely accessible RNA Sequencing data as well as clinical data available for 235 non-FAB M3 patients of this cohort were downloaded in February 2020 from https://portal.gdc.cancer.gov/projects/TARGET-AML. Other detailed information about this cohort is available online. Only 224 patients were included into the survival and treatment-response analysis after exclusion of patients above age 18yrs (N=10) and patients with OS<1 month (N=1).

Supplementary Figures

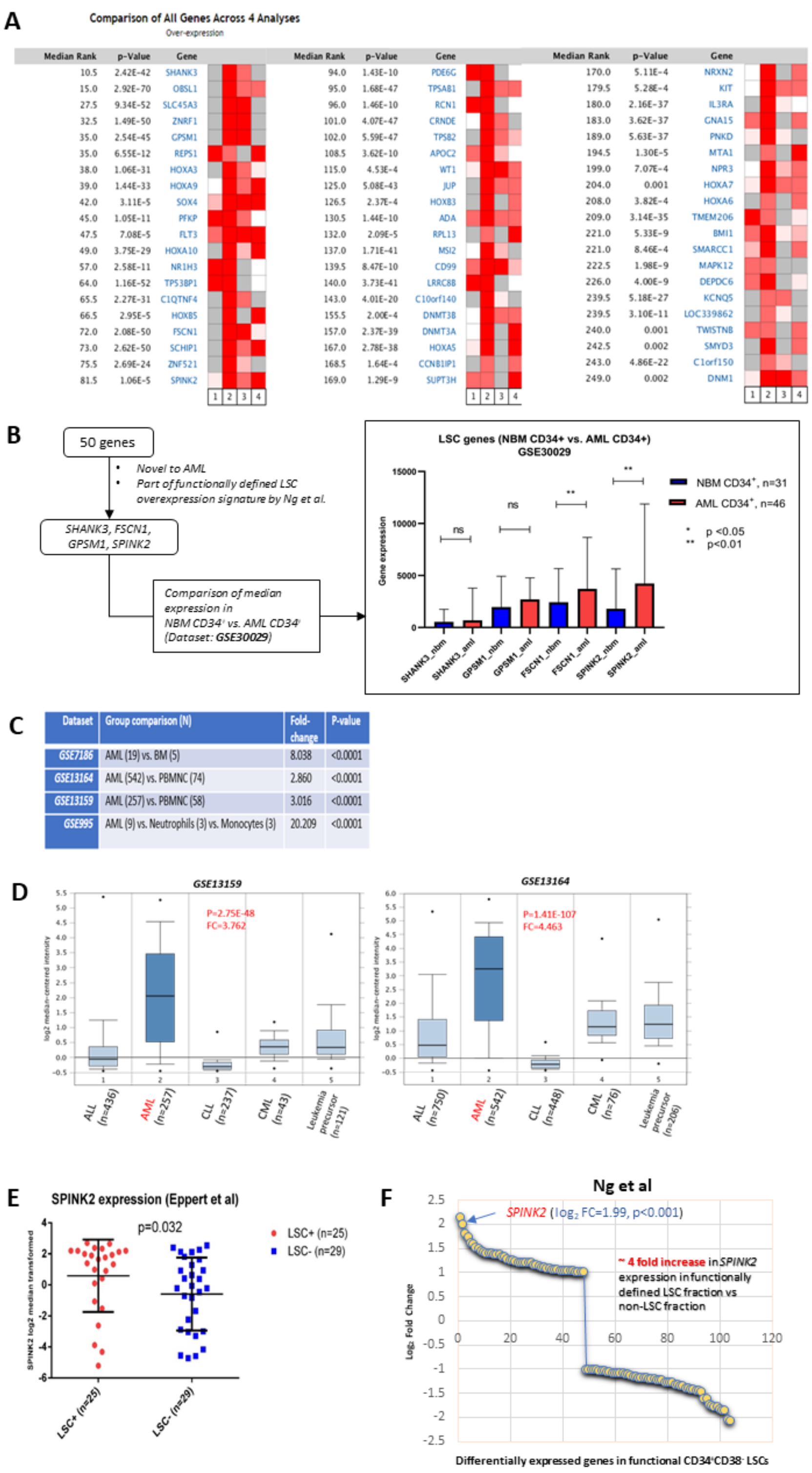

Supplementary Figure S1. Identification of *SPINK2* overexpression in AML and in LSC fractions. (A) Figure adapted from Oncomine listing the top-50 genes identified in the differential analysis comparing 831 AML samples vs. 141 NBM samples in 4 datasets – (1) Andersson (GSE7186), (2) –

Haferlach (GSE13164), (3) Haferlach 2 (GSE13159), (4) Stegmaier (GSE995), showing the genes listed according to their median rank. Grey squares indicate that the gene was not detected in the corresponding dataset.

(B) Selection criteria to filter out LSC-associated genes (*SHANK3, GPSM1, FSCN1, SPINK2*) with bar graph showing median and expression ranges in NBM $CD34^+$ and AML $CD34^+$ cells in the GSE30029 dataset. Ns – not significant. *P*-value computed by Mann-Whitney test.

(C) Data adapted from Oncomine showing the fold-change of *SPINK2* in each of the 4 datasets. In brackets are the sample sizes for each comparison. Abbreviations: BM – bone marrow, PBMNC – peripheral blood mononuclear cells

(D) Figure adapted from Oncomine demonstrating comparative analyses of *SPINK2* expression among the leukemias in datasets GSE13159 and GSE13164 which showed high expression in AML relative to other leukemias. Abbreviations: BM (bone marrow), PBMNC (peripheral blood mononuclear cells), FC (Fold Change), ALL (acute lymphoblastic leukemia), CLL (chronic lymphocytic leukemia), CML (chronic myeloid leukemia).

(E) Analysis of the Eppert *et al* dataset (GSE30377) comparing *SPINK2* expression in functionally defined leukemogenic (LSC+, n=25) or non-leukemogenic (LSC-, n=29) AML fractions (Median: 1.653 vs. -0.2122, p=0.032)

(F) Figure adapted from the Ng *et al* dataset showing the $\log_2$ foldchange of the 104 most differentially expressed genes (represented as yellow spheres) between the functionally defined LSC fraction (n=138) and non-LSC fraction (n=89). Blue arrow points to *SPINK2* at position 2 with an approximate fold-change of 4 in the LSC^+ vs. LSC^- fraction.

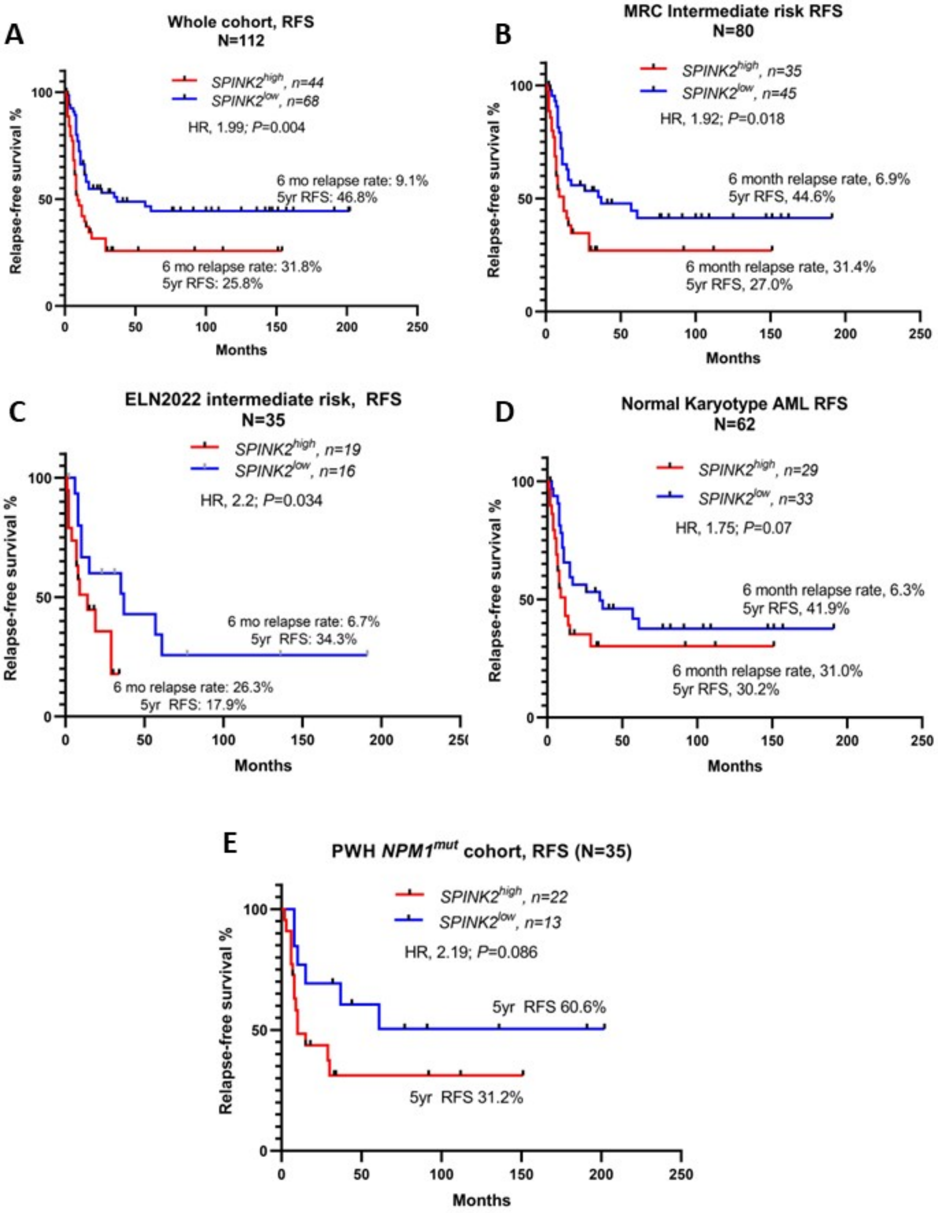

Supplementary Figure S2. Univariate Kaplan-Meier (KM) survival analysis for RFS in the whole PWH cohort (N=112), the cytogenetic IR-AML (N=80), ELN2022 IR subgroup (N=35), normal karyotype subgroup (N=62) and the *NPM1^{mut}* cohort (N=35).

(A) KM curves for RFS in the heterogeneous cohort. Median RFS in *SPINK2^{high}* vs. *SPINK2^{low}* groups: 9 vs. 37 months.

(B) KM curve for RFS in the cytogenetic IR-AML cohort. Median RFS in *SPINK2^{high}* vs. *SPINK2^{low}* groups: 12 vs. 37 months.

(C) KM curve for RFS in the ELN2022 IR-AML cohort. Median RFS in *SPINK2^{high}* vs. *SPINK2^{low}* groups: 14 vs. 37 months.

(D) KM curve for RFS in normal karyotype cohort. Median RFS in *SPINK2^{high}* vs. *SPINK2^{low}* groups: 12 vs. 35 months.

(E) KM curve for RFS in the *NPM1^{mut}* cohort. Median RFS in *SPINK2^{high}* vs. *SPINK2^{low}* groups: 14 months vs. undefined.

Survival proportions were compared using the logrank *P*-value and logrank hazard ratio (HR).

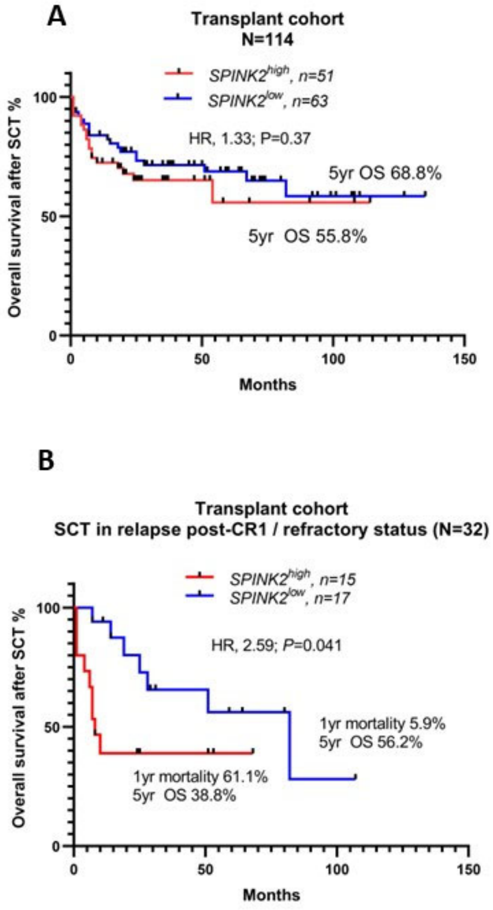

Supplementary Figure S3. ***SPINK2*** **and outcome of SCT recipients of our transplant cohort and the TCGA-LAML**

(A) KM survival curve comparing post-SCT-OS between patients with high and low median *SPINK2* expression of our combined transplant cohort, N=114. Median post-SCT OS (high vs. low *SPINK2*): both unreached

(B) KM survival curve comparing post-SCT OS between patients with high and low median *SPINK2* expression who received SCT in relapse after CR or in refractory status. Median post-SCT OS (high vs. low *SPINK2*): 8 vs. 82 months.

The logrank *P*-value and logrank hazard ratio (HR) were used for comparison of groups.

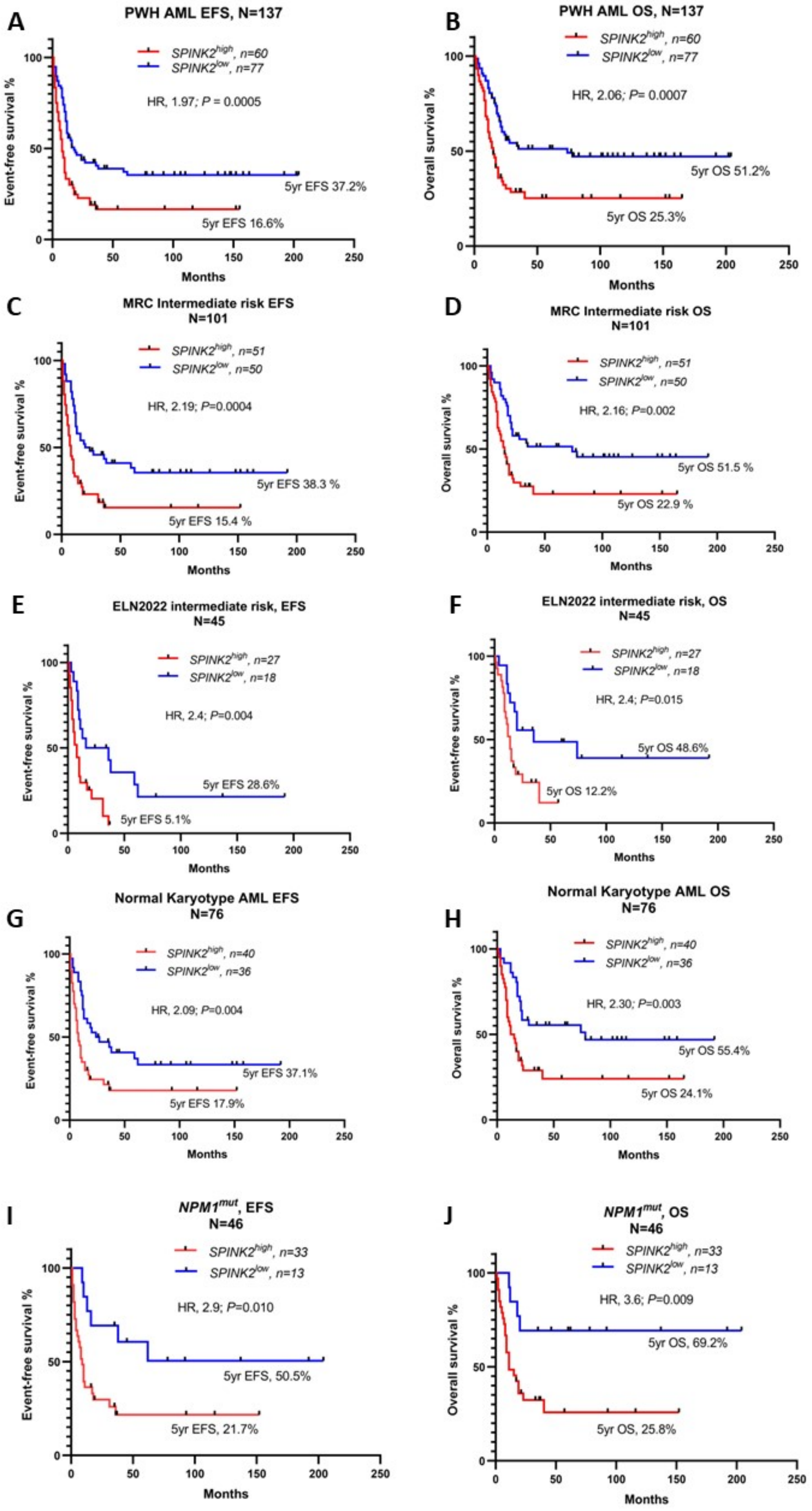

Supplementary Figure S4. Univariate KM survival analysis for EFS and OS in the heterogeneous cohort and IR, NK-AML and NPM1mut subgroups of the PWH adult AML cohort.

(A-B) Survival curves of the heterogeneous cohort (N=137) for EFS (A) and OS (B). Median survival in *SPINK2*^{high} vs. *SPINK2*^{low} groups: EFS, 8 vs 18 months; OS, 15 vs. 74 months.
(C-D) Survival curves of the cytogenetic IR cohort (N=101) for EFS (C) and OS (D). Median survival in *SPINK2*^{high} vs. *SPINK2*^{low} groups: EFS, 8 vs 22 months; OS, 15 vs. 74 months.
(E-F) Survival curves of the ELN2022 IR cohort (N=45) for EFS (E) and OS (F). Median survival in *SPINK2*^{high} vs. *SPINK2*^{low} groups: EFS, 8 vs 26 months; OS, 14 vs. 35 months.
(G-H) Survival curves of the NK-AML cohort (N=76) for EFS (G) and OS (H). Median survival in *SPINK2*^{high} vs. *SPINK2*^{low} groups: EFS, 8 vs 25.5 months; OS, 13.5 vs. 78 months.
(I-J) Survival curves of the NPM1^{mut} cohort (N=46) for EFS (I) and OS (J). Median survival in *SPINK2*^{high} vs. *SPINK2*^{low} groups: EFS, 9 months vs unreached; OS, 11 months vs. unreached.
Survival proportions were compared using the logrank *P*-value and logrank hazard ratio (HR).

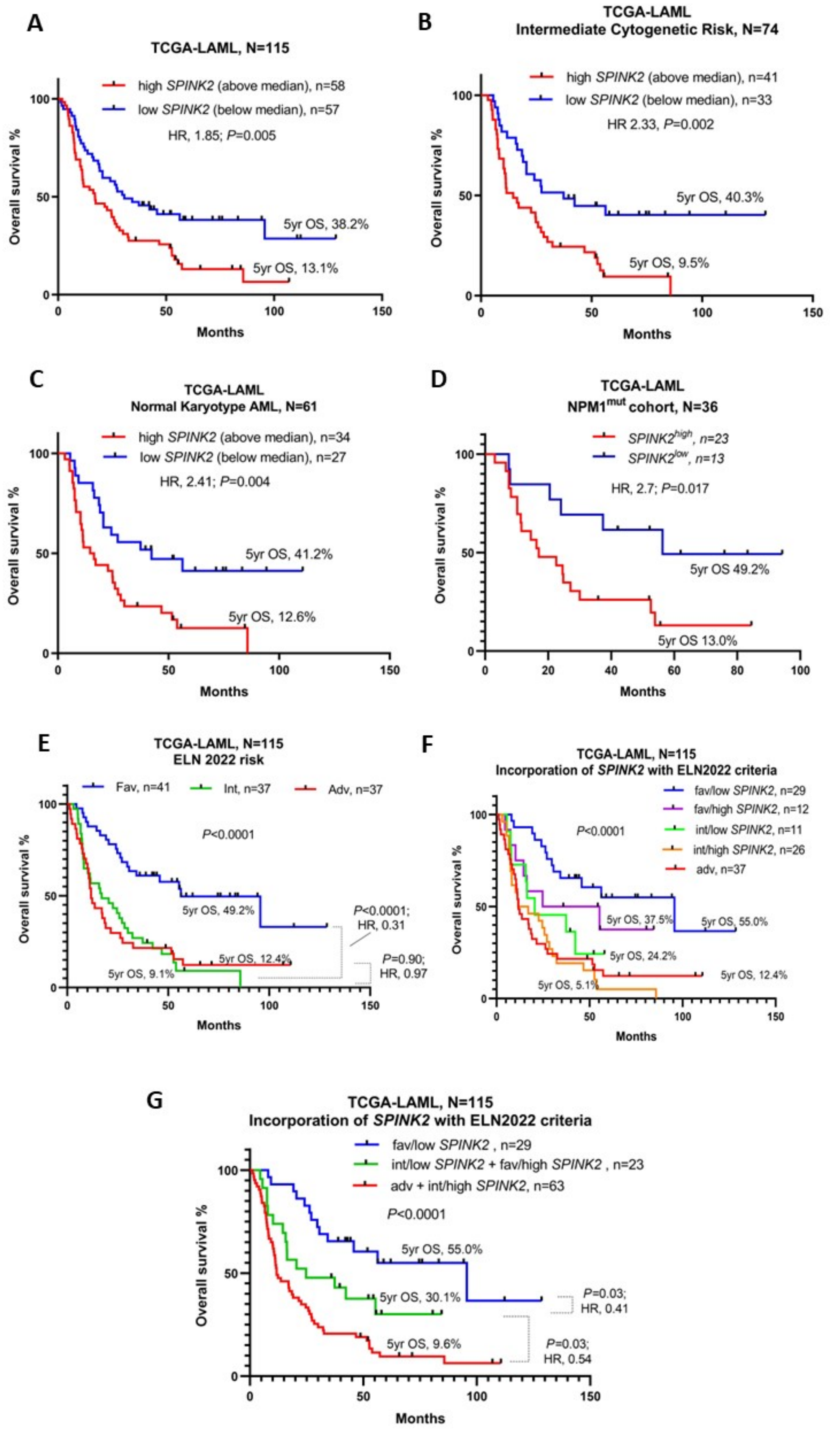
A
TCGA-LAML, N=115
high SPINK2 (above median), n=58
low SPINK2 (below median), n=57
HR, 1.85; P=0.005
5yr OS, 38.2%
5yr OS, 13.1%
Overall survival %
Months
B
TCGA-LAML
Intermediate Cytogenetic Risk, N=74
high SPINK2 (above median), n=41
low SPINK2 (below median), n=33
HR 2.33, P=0.002
5yr OS, 40.3%
5yr OS, 9.5%
Overall survival %
Months
C
TCGA-LAML
Normal Karyotype AML, N=61
high SPINK2 (above median), n=34
low SPINK2 (below median), n=27
HR, 2.41; P=0.004
5yr OS, 41.2%
5yr OS, 12.6%
Overall survival %
Months
D
TCGA-LAML
NPM1mut cohort, N=36
SPINK2high, n=23
SPINK2low, n=13
HR, 2.7; P=0.017
5yr OS 49.2%
5yr OS 13.0%
Overall survival %
Months
E
TCGA-LAML, N=115
ELN 2022 risk
Fav, n=41
Int, n=37
Adv, n=37
P<0.0001
5yr OS, 49.2%
P<0.0001; HR, 0.31
5yr OS, 12.4%
5yr OS, 9.1%
P=0.90; HR, 0.97
Overall survival %
Months
F
TCGA-LAML, N=115
Incorporation of SPINK2 with ELN2022 criteria
fav/low SPINK2, n=29
fav/high SPINK2, n=12
int/low SPINK2, n=11
int/high SPINK2, n=26
adv, n=37
P<0.0001
5yr OS, 37.5%
5yr OS, 55.0%
5yr OS, 24.2%
5yr OS, 12.4%
5yr OS, 5.1%
Overall survival %
Months
G
TCGA-LAML, N=115
Incorporation of SPINK2 with ELN2022 criteria
fav/low SPINK2 , n=29
int/low SPINK2 + fav/high SPINK2 , n=23
adv + int/high SPINK2, n=63
P<0.0001
5yr OS, 55.0%
5yr OS, 30.1%
P=0.03; HR, 0.41
5yr OS, 9.6%
P=0.03; HR, 0.54
Overall survival %
Months

Supplementary Figure S5. Univariate survival analyses (OS) of *SPINK2* mRNA overexpression in the TCGA-LAML whole cohort and indicated subgroups.

(A) Kaplan-Meier (KM) survival curves for OS in the heterogeneous cohort including all ages (N=115). Median OS (high vs low *SPINK2*): 17.1 vs 30.6 months.

(B) KM survival curve for OS in the cytogenetic IR subgroup: Median OS (high vs low *SPINK2*) – 14.5 vs 37.4 months.

(C) KM survival curve for OS in the normal karyotype subgroup: Median OS (high vs low *SPINK2*) – 15.4 vs 42.3 months.

(D) KM survival curve for OS in the *NPM1*^{mut} subgroup: Median OS (high vs low *SPINK2*) – 17.0 vs 56.3 months.

(E) KM survival curve for OS categorized by ELN2022 risk criteria only. Median OS (fav vs. int vs. adv risk) – 56.3 vs. 17 vs 11.8 months. Logrank trend test, $P<0.0001$.

(F) KM survival curve for OS categorized by ELN2022 risk criteria with incorporation of SPINK2 expression status. Logrank trend test, $P<0.0001$.

(G) KM survival curve for OS categorized by ELN2022 risk criteria with incorporation of SPINK2 expression by combining categories as indicated. Median OS (adv+int/high SPINK2 vs. int/low SPINK2 + fav/high SPINK2 vs. fav/low SPINK2) –11.8 vs. 24.8 vs. 95.6 months. Logrank trend test, $P<0.0001$.

Survival proportions were compared using the logrank *P*-value and logrank hazard ratio (HR).

Abbreviations: Fav, favorable risk; Int, intermediate risk; Adv, adverse risk.

A

Percentile	*SPINK2* FC	OS			EFS		
		P-value	HR	HR (95% C.I.)	*P*-value	HR	HR (95% C.I.)
10th Percentile	0.002544	0.9514	1.046	0.2526 - 4.331	0.9758	1.019	0.3130 - 3.314
20th Percentile	0.07271	0.3068	1.858	0.6954 - 4.963	0.3741	1.535	0.6663 - 3.537
30th Percentile	0.1199	0.116	2.305	0.9745 - 5.452	0.1855	1.747	0.8354 - 3.653
40th Percentile	0.4812	0.1014	2.124	0.9431 - 4.786	0.1584	1.7	0.8453 - 3.419
50th Percentile	1.269	0.3169	1.508	0.6771 - 3.358	0.6327	1.183	0.5904 - 2.372
60th Percentile	8.881	0.0548	2.149	0.9312 - 4.958	0.0809	1.83	0.8736 - 3.834
**70th Percentile**	**13.57**	**0.0127**	**2.644**	**1.079 - 6.482**	**0.0055**	**2.56**	**1.128 - 5.811**
80th Percentile	30.85	0.1494	1.884	0.6639 - 5.344	0.0285	2.279	0.8555 - 6.070
90th Percentile	112.5	0.0068	3.528	0.7310 - 17.03	0.0067	3.418	0.7116 - 16.41

Patient dichotomization at 70th percentile

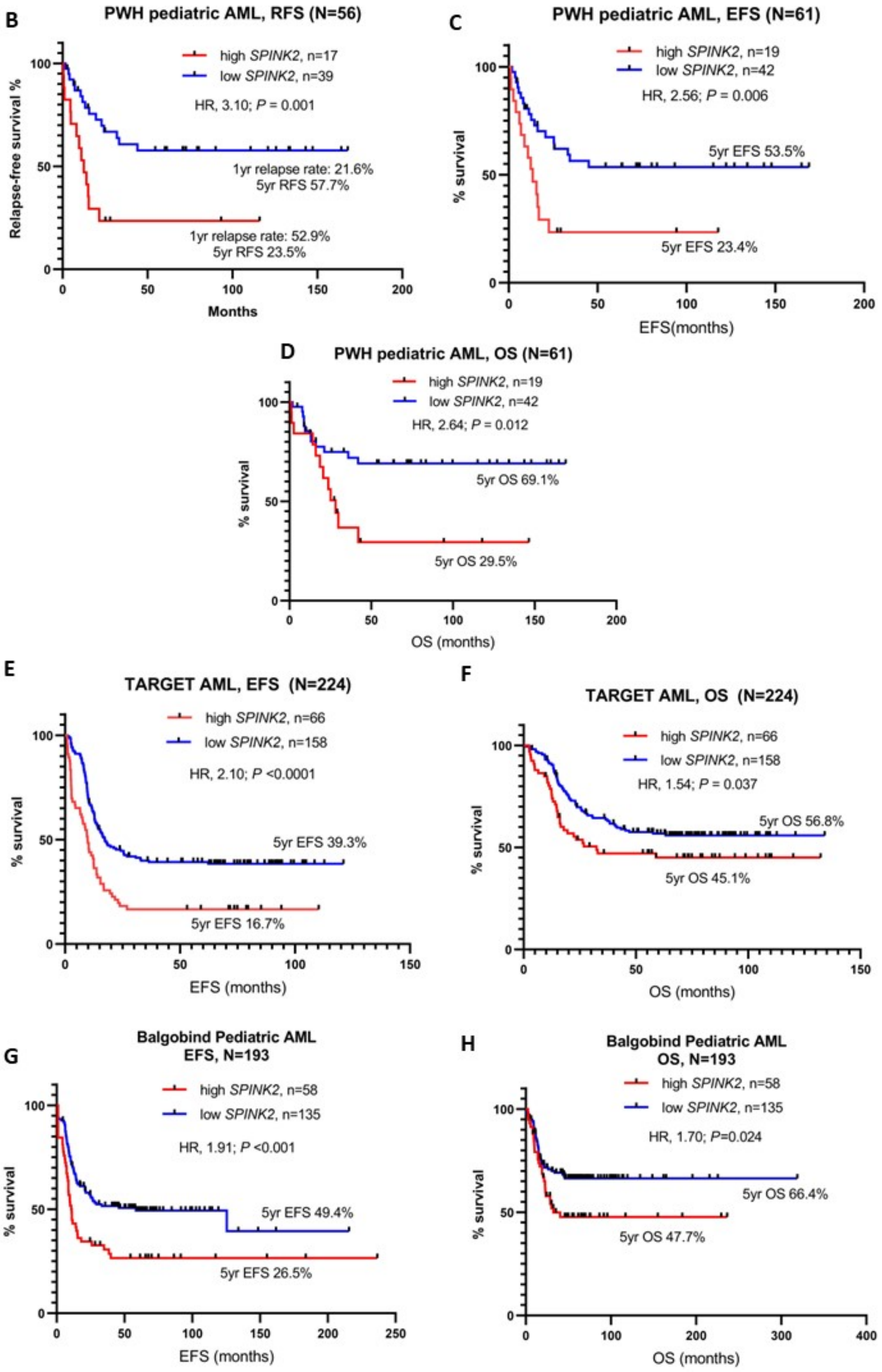

Supplementary Figure S6. SPINK2 and pediatric AML: Univariate survival analysis in the PWH pediatric AML cohort (N=61) and in the TARGET-AML (N=224) and Balgobind (N=193) pediatric AML datasets.

(A) In order to determine an optimal expression cut-off with strongest prognostic implications, the cohort of 61 patients was first examined by univariate Cox survival analysis comparing OS and EFS using 10% increments of SPINK2 expression fold-change (FC). A cut-off at the 70th percentile demonstrated strongest association with adverse outcome in terms of the log-rank *P*-value, hazard ratio (HR) and 95% CI of HR. The subsequent Kaplan Meier (KM) survival and clinicopathological analyses were performed by dichotomization of the cohort at this cut-off.

(B) KM survival curve for RFS in pediatric AML patients who had achieved CR: Median RFS (high vs low *SPINK2*) – 12.5 months vs unreached.

(C) KM survival curve for EFS in pediatric AML patients: Median EFS (high vs low *SPINK2*) – 13.5 months vs unreached.

(D) KM survival curve for OS in in pediatric AML patients: Median OS (high vs low *SPINK2*) – 28.1 months vs unreached.

(E) KM survival curve for EFS in the TARGET AML cohort: Median EFS (high vs low *SPINK2*) – 9.9 vs 17.3 months.

(F) KM survival curve for OS in the TARGET AML cohort: Median OS (high vs low *SPINK2*) – 32.5 months vs unreached.

(G) KM survival curve for EFS in the Balgobind cohort: Median EFS (high vs low *SPINK2*) – 11.0 vs 58.4 months.

(H) KM survival curve for OS in the Balgobind cohort: Median OS (high vs low *SPINK2*) – 32.3 months vs unreached.

Survival proportions were compared using the logrank *P*-value and logrank hazard ratio (HR).

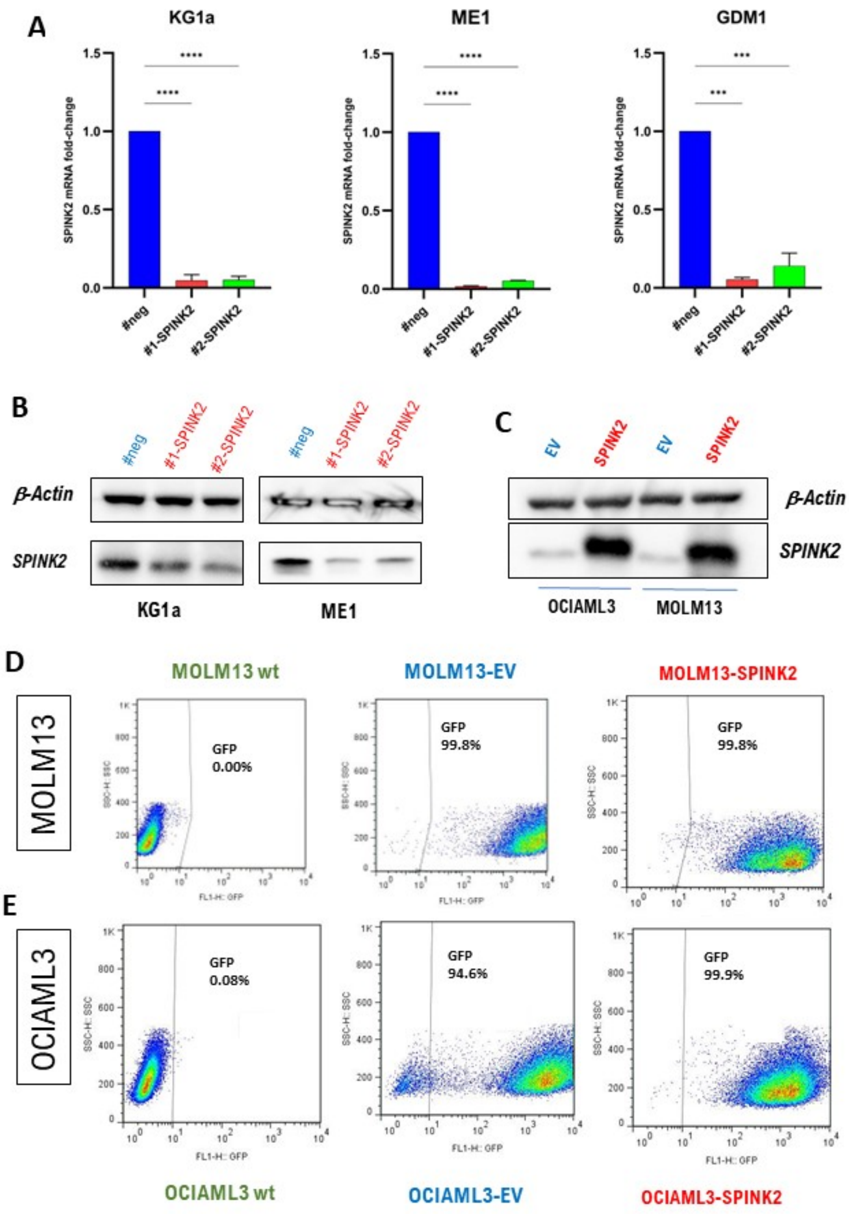

Supplementary Figure S7. Modulation of *SPINK2* gene expression in KG1a, ME1, GDM1, MOLM13 and OCIAML3 cells.

(A) qPCR confirming the reduction of *SPINK2* expression upon *SPINK2* knockdown with siRNAs #1 and #2 in KG1a (left), ME1 (centre) and GDM1 (right) cells. qPCR shows the mean ± SD of at least 3 independent experiments in KG1a and ME1 cells, and the mean ± SD of 2 independent experiments in GDM1. Statistics, ordinary one-way ANOVA and Dunnett's multiple comparisons test. *** $P<0.001$, ****$P<0.0001$ (B) Western blots showing decrease in *SPINK2* protein expression in cells with SPINK2 knockdown. β-ACTIN was used as loading control. (C) Western Blot of *SPINK2* in OCIAML3 and MOLM13 cells transduced with empty vector (EV) and *SPINK2* lentiviruses. β-ACTIN was used as loading control. (D-E) Flow cytometry showing transduction efficiency in MOLM13 (D) and OCIAML3 (E) cells as measured by percentage of GFP^+ cells after 1 week of puromycin selection at 1μg/ml. Transduction efficiency was consistently >90%.

Supplementary references

[1] D.R. Rhodes, J. Yu, K. Shanker, N. Deshpande, R. Varambally, D. Ghosh, T. Barrette, A. Pandey and A.M. Chinnaiyan, '"ONCOMINE: a cancer microarray database and integrated data-mining platform," *Neoplasia*, vol. 6, no. 1, Jan, pp. 1-6.

[2] S.W.K. Ng, A. Mitchell, J.A. Kennedy, W.C. Chen, J. McLeod, N. Ibrahimova, A. Arruda, A. Popescu, V. Gupta, A.D. Schimmer, A.C. Schuh, K.W. Yee, L. Bullinger, T. Herold, D. Görlich, T. Büchner, W. Hiddemann, W.E. Berdel, B. Wörmann, M. Cheok, C. Preudhomme, H. Dombret, K. Metzeler, C. Buske, B. Löwenberg, P.J.M. Valk, P.W. Zandstra, M.D. Minden, J.E. Dick and J.C.Y. Wang, '"A 17-gene stemness score for rapid determination of risk in acute leukaemia," *Nature*, vol. 540, no. 7633, pp. 433-437.

[3] H.J.M. de Jonge, C.M. Woolthuis, A.Z. Vos, A. Mulder, d.B. van, P.M. Kluin, d.W. van, de Bont, E. S. J. M., G. Huls, E. Vellenga and J.J. Schuringa, '"Gene expression profiling in the leukemic stem cell-enriched CD34+ fraction identifies target genes that predict prognosis in normal karyotype AML," *Leukemia*, vol. 25, no. 12, pp. 1825-1833.

[4] K. Eppert, K. Takenaka, E.R. Lechman, L. Waldron, B. Nilsson, P. van Galen, K.H. Metzeler, A. Poeppl, V. Ling, J. Beyene, A.J. Canty, J.S. Danska, S.K. Bohlander, C. Buske, M.D. Minden, T.R. Golub, I. Jurisica, B.L. Ebert and J.E. Dick, '"Stem cell gene expression programs influence clinical outcome in human leukemia," *Nat.Med.*, vol. 17, no. 9, pp. 1086-1093.

[5] H. Döhner, A.H. Wei, F.R. Appelbaum, C. Craddock, C.D. DiNardo, H. Dombret, B.L. Ebert, P. Fenaux, L.A. Godley, R.P. Hasserjian, R.A. Larson, R.L. Levine, Y. Miyazaki, D. Niederwieser, G. Ossenkoppele, C. Röllig, J. Sierra, E.M. Stein, M.S. Tallman, H. Tien, J. Wang, A. Wierzbowska and B. Löwenberg, '"Diagnosis and management of AML in adults: 2022 recommendations from an international expert panel on behalf of the ELN," *Blood*, vol. 140, no. 12, pp. 1345-1377.

[6] Anonymous '"Genomic and Epigenomic Landscapes of Adult De Novo Acute Myeloid Leukemia," *N.Engl.J.Med.*, vol. 368, no. 22, pp. 2059-2074.

[7] A.J. Gentles, S.K. Plevritis, R. Majeti and A.A. Alizadeh, '"Association of a leukemic stem cell gene expression signature with clinical outcomes in acute myeloid leukemia," *JAMA*, vol. 304, no. 24, Dec 22, pp. 2706-2715.

[8] J.W. Tyner, C.E. Tognon, D. Bottomly, B. Wilmot, S.E. Kurtz, S.L. Savage, N. Long, A.R. Schultz, E. Traer, M. Abel, A. Agarwal, A. Blucher, U. Borate, J. Bryant, R. Burke, A. Carlos, R. Carpenter, J. Carroll, B.H. Chang, C. Coblentz, A. d'Almeida, R. Cook, A. Danilov, K.T. Dao, M. Degnin, D. Devine, J. Dibb, D.K. Edwards, C.A. Eide, I. English, J. Glover, R. Henson, H. Ho, A. Jemal, K. Johnson, R. Johnson, B. Junio, A. Kaempf, J. Leonard, C. Lin, S.Q. Liu, P. Lo, M.M. Loriaux, S. Luty, T. Macey, J. MacManiman, J. Martinez, M. Mori, D. Nelson, C. Nichols, J. Peters, J. Ramsdill, A. Rofelty, R. Schuff, R. Searles, E. Segerdell, R.L. Smith, S.E. Spurgeon, T. Sweeney, A. Thapa, C. Visser, J. Wagner, K. Watanabe-Smith, K. Werth, J. Wolf, L. White, A. Yates, H. Zhang, C.R. Cogle, R.H. Collins, D.C. Connolly, M.W. Deininger, L. Drusbosky, C.S. Hourigan, C.T. Jordan, P. Kropf, T.L. Lin, M.E. Martinez, B.C. Medeiros, R.R. Pallapati, D.A. Pollyea, R.T. Swords, J.M. Watts, S.J. Weir, D.L. Wiest, R.M. Winters, S.K. McWeeney and B.J. Druker, '"Functional genomic landscape of acute myeloid leukaemia," *Nature*, vol. 562, no. 7728, pp. 526-531.

[9] R.G. Verhaak, B.J. Wouters, C.A. Erpelinck, S. Abbas, H.B. Beverloo, S. Lugthart, B. Löwenberg, R. Delwel and P.J. Valk, '"Prediction of molecular subtypes in acute myeloid leukemia based on gene expression profiling," *Haematologica*, vol. 94, no. 1, Jan, pp. 131-134.

[10] B.V. Balgobind, M.M. Van den Heuvel-Eibrink, R.X. De Menezes, D. Reinhardt, I.H. Hollink, S.T. Arentsen-Peters, E.R. van Wering, G.J. Kaspers, J. Cloos, E.S. de Bont, J.M. Cayuela, A. Baruchel, C. Meyer, R. Marschalek, J. Trka, J. Stary, H.B. Beverloo, R. Pieters, C.M. Zwaan and M.L. den Boer, '"Evaluation of gene expression signatures predictive of cytogenetic and molecular subtypes of pediatric acute myeloid leukemia," *Haematologica*, vol. 96, no. 2, Feb, pp. 221-230.
